# The yeast ubiquitin ligase SCF^Met30^: connecting environmental and intracellular conditions to cell division

**DOI:** 10.1186/1747-1028-1-16

**Published:** 2006-08-08

**Authors:** Peter Kaiser, Ning-Yuan Su, James L Yen, Ikram Ouni, Karin Flick

**Affiliations:** 1 University of California Irvine, Department of Biological Chemistry, School of Medicine 240D Med Sci I Irvine, CA 92697-1700, USA

## Abstract

Ubiquitination regulates a host of cellular processes and is well known for its role in progression through the cell division cycle. In budding yeast, cadmium and arsenic stress, the availability of sulfur containing amino acids, and the intracellular concentration of S-adenosylmethionine are linked to cell cycle regulation through the ubiquitin ligase SCF^Met30^. Regulation is achieved by ubiquitination of the transcription factor Met4. Met4 activity is controlled by a regulatory K48-linked ubiquitin chain that is synthesized by Cdc34/SCF^Met30^. A ubiquitin-interacting-motif (UIM) present in Met4 prevents degradation of ubiquitinated Met4 allowing the ubiquitin chain to function as a reversible switch of Met4 activity. Here we discuss mechanisms of Met4 and SCF^Met30 ^regulation in response to intracellular and environmental conditions, and describe the integration of these signals with cell cycle control.

## Review

**Figure 1:**
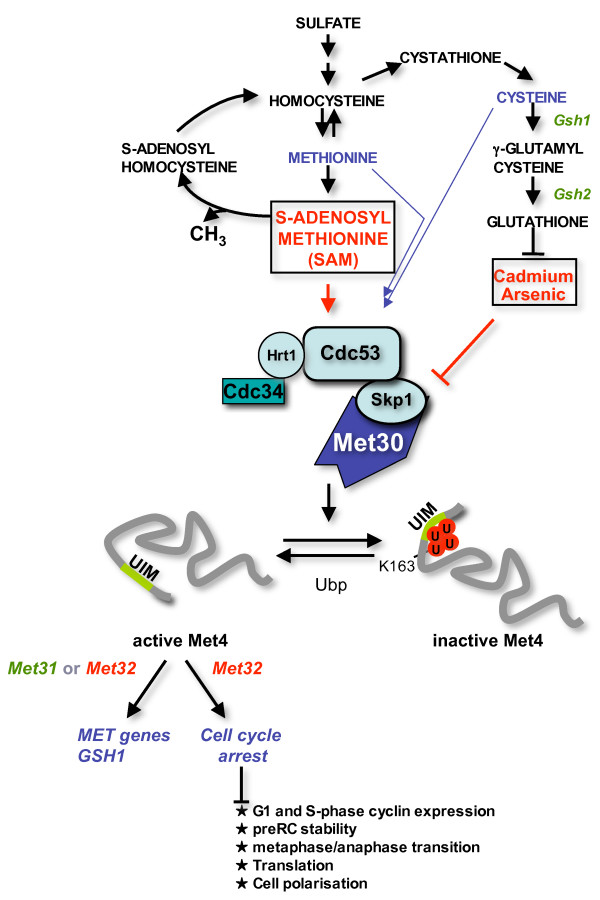
**Regulation of the ubiquitin ligase SCF^Met30 ^and its connection to cell cycle control**. SCF^Met30 ^ubiquitinates the transcriptional activator Met4 on lysine 163 to maintain it in an inactive state. Degradation of poly-ubiquitinated Met4 is prevented by an ubiquitin-interacting motif (UIM) in Met4. The sulfur containing compounds methionine, cysteine, and S-adenosylmethionine stimulate Met4 ubiquitination, but when their intracellular concentrations are low, reduced ubiquitination combined with Met4 deubiquitination by a so far unidentified deubiquitinating enzyme(s) (Ubp) leads to Met4 activation. Cadmium and arsenic stress inhibit Met4 ubiquitination by inactivation of the ubiquitin ligase SCF^Met30^. The resulting activation of Met4 raises cellular glutathione levels due to induced expression of Gsh1. Active Met4 induces expression of a number of genes involved in the sulfur amino acid synthesis pathway (*MET*-genes) as well as *GSH1*. *MET*-gene expression depends on either one of the two homologous zinc-finger proteins Met31 and Met32. Met4 activation also inhibits a number of cell cycle events and can lead to cell cycle arrest. The Met4-dependent cell cycle arrest requires Met32 but cannot be mediated by Met31.

As indicated by its name Met30 was initially identified as a component involved in regulation of the methionine biosynthesis pathway in budding yeast [1]. Based on initial genetic studies, Met30 was proposed to be required for inactivation of the transcriptional activator Met4, which functions as the master regulator of the transcription network that coordinates synthesis of sulfur containing molecules such as methionine, cysteine and S-adenosylmethionine (Fig. 1) [2,3]. Met30 has also been shown to be essential for cell cycle progression and thus connects the cell cycle with metabolism [4-6]. A possible molecular function was suggested by the identification of an F-box motif in Met30 [7], which linked Met30 to the ubiquitin/proteasome pathway. Proteins with F-box motifs generally function as substrate adaptors in SCF ubiquitin ligases and mediate substrate specificity of ubiquitin ligase complexes [8]. Met30 was subsequently shown to form the ubiquitin ligase SCF^Met30^, which is essential for viability [1,4,9]. Genetic results indicate that SCF^Met30 ^controls the activity of the transcription factor Met4. Active Met4 induces genes involved in the synthesis of important sulfur containing metabolites (methionine, cysteine, S-adenosylmethionine) [10,11] and triggers the cellular response to cadmium and arsenic exposure [12,13]. Full activation of Met4 will also arrest cell proliferation at several positions in the cell cycle [5,6], and has been proposed to inhibit phosphatidylserine transport to mitochiondria ([14,15] and personal communication D.R. Voelker).

In the following sections we will discuss regulation of Met4 and SCF^Met30 ^activity, the Met4-induced cell cycle arrests and the biological significance of the SCF^Met30^/Met4 pathway.

## Regulation of the transcription factor Met4 by SCF^Met30^

**Figure 2:**
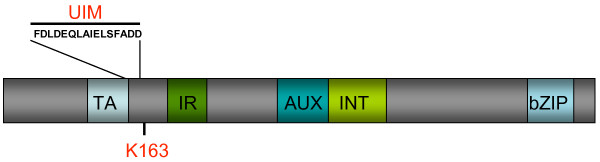
**Met4 domain structure**. TA: transactivating domain. IR: inhibitory region required for repression of Met4 activity by methionine. AUX: auxiliary domain required to fully relieve IR-mediated repression under activating conditions. INT: protein-protein interaction domain that binds Met31 and Met32. bZIP: basic leucine zipper domain that mediates Met4 dimerization and binding to Met28 as well as Cbf1. UIM: ubiquitin-interacting motif that prevents degradation of poly-ubiquitinated Met4. K163: ubiquitin acceptor site.

The genetic observation that deletion of *MET4 *can bypass the cell cycle requirement for Met30 indicated that Met4 can inhibit cell proliferation and that Met30 is necessary to prevent Met4 from inducing cell cycle arrest [5,10]. Consistent with the genetic results, *in vivo *and *in vitro *experiments demonstrated that SCF^Met30 ^ubiquitinates Met4 [10,13]. Unexpectedly, ubiquitination of Met4 does not induce its degradation by the proteasome. Instead, the attached ubiquitin chain has a direct regulatory role and inhibits Met4 activity [10,16-18]. It is important to note that under certain growth conditions the strictly regulatory and non-proteolytic function of the Met4-attached ubiquitin chain can be transformed into a degradation signal to induce Met4 degradation by the 26S proteasome [17,18]. The specific conditions responsible for this change in ubiquitin chain function remain to be identified. In addition, the biological significance of the Met4 degradation pathway is not clear because non-proteolytic ubiquitination is sufficient for Met4 inactivation [10,17,18]. The biological advantage of non-proteolytic regulation by ubiquitination over proteolytic regulation of Met4 has recently been demonstrated. The non-proteolytic pathway enables cells to maintain a stable but inactive pool of Met4 that can be rapidly activated by deubiquitination to trigger a fast cellular response [16]. In contrast, proteolytic regulation results in significantly delayed Met4 activation due to the requirement for protein synthesis to build up a significant pool of Met4 [16]. The same study also described how ubiquitinated Met4 escapes degradation. A short ubiquitin-binding domain, similar to the ubiquitin-interacting-motif (UIM) found in several ubiquitin-binding proteins [19], is located in the N-terminal region of Met4 (Fig. 2) [16]. The UIM in Met4 binds to the Met4-attached ubiquitin chain in *cis*. This interaction protects the ubiquitin chain from the proteasome and also appears to prevent addition of further ubiquitin units by Cdc34/SCF^Met30 ^to restrict ubiquitin chain length (Fig. 1) [16]. Remarkably, mutations in conserved residues of the UIM in Met4 that block UIM binding to ubiquitin chains, transform Met4 into an unstable protein. Furthermore, mutating the UIM allows the assembly of a longer ubiquitin chain on Met4 [16].

The ubiquitin chain attached to Met4 was itself the subject of detailed studies [17]. Cdc34/SCF^Met30 ^assembles the chain on a single lysine residue in Met4. Blocking Met4 ubiquitination by mutating this lysine acceptor site (lysine in position 163 mutated to arginine) results in a constitutively active Met4, which provids direct evidence for a regulatory role of Met4 ubiquitination [17]. Ubiquitin chains can be assembled by ubiquitin-ubiquitin linkages using any one of seven different lysine residues in ubiquitin [20,21]. Analyses of the ubiquitin chain topology *in vivo *revealed that Met4 is modified by a homogenous ubiquitin chain that is linked through the lysine in position 48 in ubiquitin (lys48-chain) [17]. Lys48-linked ubiquitin chains serve as the predominant signal for degradation by the proteasome [22]. Interestingly, the UIM motif in Met4 shows binding preference for lys48 chains further demonstrating that the UIM/ubiquitin interaction masks a *bona fide *degradation signal [16].

Direct inhibition of Met4 activity by regulatory ubiquitination of lysine in position 163 has been clearly established [17]. The detailed mechanism of how ubiquitination blocks transactivation by Met4 is currently unknown. Met4 ubiquitination has been correlated with loss of promoter recruitment [18] and changes in protein-protein interactions [10], but the molecular determinants of this regulation remain to be identified. Notably, the UIM domain that is essential to prevent degradation of ubiquitinated Met4 is not required for Met4 inactivation as ubiquitination of Met4 without a functional UIM domain is sufficient to completely inactivate Met4 even when overproduced to compensate for reduced Met4 levels ([16] and Flick K. unpublished observation).

## SCF^Met30^-dependent cell cycle steps

The essential cell cycle function of SCF^Met30 ^is mediated through Met4 [5,10]. Studies using a ubiquitin acceptor site mutant Met4 (Met4^K163R^) provided direct evidence that fully deubiquitinated Met4 functions as a cell cycle inhibitor [17]. Both, the transactivation and the so-called INT domain of Met4 (Fig. 2) are essential for induction of cell cycle arrest [5]. The INT domain forms a protein interaction site for the two homologous zinc-finger proteins Met31 and Met32 (Fig. 2) [23]. Consistent with the requirement of the INT domain for cell cycle arrest, deletion of *MET32*, like deletion of *MET4*, bypasses the lethality of *met30 *mutants [5]. Interestingly, although Met31 function is largely redundant with Met32 in *MET-*gene expression, deletion of Met31 cannot suppress *met30 *mutants [5]. These genetic results suggest that a Met4/Met32 complex regulates expression of thus far unidentified genes that induce cell cycle arrest.

Cell cycle experiments using both a conditional system that relies on galactose-induced expression of Met4 in *met30 *mutants and temperature sensitive *met30 *mutants revealed a surprisingly complex picture of Met30-dependent cell cycle regulation (Fig. 1). Most of the cell cycle studies have focused on the G_1_-S transition function of Met30, the primary cell cycle arrest position observed in *met30 *mutants [4-6]. Studies with presynchronized cells showed that expression of the G_1 _cyclins *CLN1 *and *CLN2 *and the S-phase cyclin *CLB5 *depend on Met30 function. In contrast, expression of *CLN3*, the third G_1 _cyclin, is unaffected by loss of Met30 function [5,6]. Although the cyclin expression defects affect G_1_-S transition, they cannot explain the arrest phenotype of *met30 *mutants, because neither overproduction of Cln2 nor Clb5 from a heterologous promoter can drive *met30 *mutants into S-phase. Similarly, deletion of the S-phase inhibitor Sic1, which imposes the requirement for G_1 _cyclin-dependent kinase activity on S-phase entry, does not suppress the cell cycle defect of *met30 *mutants [6]. These results suggest that additional G_1_-S phase steps depend on Met30. Indeed, chromatin-immunoprecipitation experiments indicate that Met30 is important to maintain pre-replication complexes (pre-RC) at origins of replications [6]. Consistent with these results is the observation that Met30 function is completely dispensable for S-phase once pre-replication complexes have been activated and are no longer required for S-phase initiation [5,6].

In addition to cyclin expression and pre-RC stability at origins, Met30 has been implicated in actin polarization, metaphase-anaphase transition [6], protein synthesis [5], and degradation of Swe1, the budding yeast homolog of the cell cycle inhibiting kinase Wee1 [4]. SCF^Met30 ^is probably not directly involved in Swe1 degradation because deletion of *MET4 *suppresses the Swe1 degradation defect of *met30 *mutants [24]. This is somewhat surprising considering that the mammalian relatives of SCF^Met30^, SCF^β-TRCP1/2^, function as ubiquitin ligases for mammalian Wee1 [25].

A role for Met30 in protein synthesis is evident in cells experiencing extended periods of Met30 depletion but is unlikely to contribute to the initial cell cycle arrest because both cell mass increase and protein synthesis slow down only after prolonged cell cycle arrest of *met30 *mutants [5].

Cell cycle studies have connected Met30 function to several important cell cycle steps. However, molecular mechanisms for Met30 in regulation of cyclin expression, pre-RC stability, nuclear division, actin polarization, or translation has not been reported so far. Most of the Met30 cell cycle functions are mediated by the Met4/Met32 transcription complex and are therefore likely to be executed by Met4/Met32-induced gene expression. It is important to bear in mind that SCF^Met30 ^does not play an activator role in the cell cycle, but is instead required to suppress Met4/Met32-induced cell cycle inhibition. SCF^Met30 ^can thus be viewed as an inhibitor of a complex cell cycle checkpoint response.

## SCF^Met30 ^and the cellular response to cadmium and arsenic

A first indication that SCF^Met30 ^could play a significant role in regulating the response of yeast cells to exposure to the toxic heavy metal cadmium came from the observation that Met4 activation is important for cadmium resistance [26-29]. Recently, SCF^Met30 ^and the fission yeast homolog SCF^Pof1 ^were directly linked to the cellular response to cadmium ([12,13,30], for a recent review see [31]).

**Figure 3:**
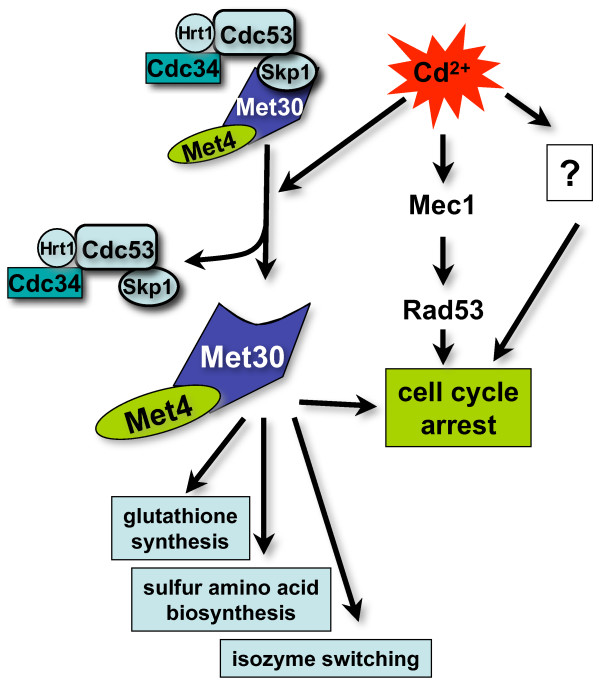
**Cadmium-induced regulation of SCF^Met30^**. Cadmium leads to dissociation of Met30 from the SCF-core by preventing the Met30/Skp1 interaction and blocks Met4 ubiquitination. The resulting activation of Met4 induces synthesis of sulfur amino acids and glutathione as well as a process called isozyme switching, which is part of the sulfur sparing response. Cadmium also leads to activation of three independent cell cycle arrest pathways. The Met4/Met32-dependend and the Mec1/Rad53-dependent pathways, as well as one so far unidentified pathway, which was indicated by genetic results.

Met4 ubiquitination is blocked in response to cadmium leading to a rapid induction of Met4-dependent gene expression [12,13]. Similar effects were observed in cells exposed to arsenic [12,32], but not to other heavy metals [12,13]. Intracellular cadmium and arsenic detoxification is primarily achieved by covalent sequestration by the tripeptide glutathione [33]. The rate-limiting step in glutathione biosynthesis is catalyzed by Gsh1 (γ-glutamyl-cysteine-synthase) (Fig. 1). Cadmium and arsenic block Met4 ubiquitination to induce a Met4-dependent transcription program, which includes induction of *GSH1 *expression [12,13,28,29], and what has been described as the "sulfur sparing response" [28,34]. Sulfur-sparing refers to the observation that upon cadmium stress, yeast cells repress expression of several glycolytic enzymes and instead strongly induce expression of isozymes with a significantly lower content of sulfur containing amino acids [28]. This isozyme switching has been proposed to help cells dedicate more of their sulfur resources to glutathione synthesis [28,31,34]. Isozyme switching could also make glycolysis less vulnerable to the toxic effects of cadmium because the induced isozymes have a markedly reduced number of sulfhydryl groups, which are the main targets for cadmium-induced protein damage [35,36] (Fig. 3).

Cadmium also induces a complex cell cycle checkpoint response [12] (Fig. 3). Not all pathways that lead to the cadmium induced cell cycle arrest have been identified, but activation of the Met4/Met32 pathway via inhibition of SCF^Met30 ^clearly plays an important role. In addition, cadmium (and arsenic) exposure activates the checkpoint kinases Mec1 and Rad53, which is likely to contribute to the G_1 _cell cycle arrest. In addition, the Mec1/Rad53 pathway is responsible for slow progression through S-phase when cells that have already initiated DNA replication experience cadmium stress [12]. Genetic experiments suggest at least one additional cadmium-induced cell cycle arrest mechanism that prevents entry into S-phase during cadmium stress conditions, because cadmium exposed *mec1 met32 *double mutants still arrest in G_1 _[12] (Fig. 3). Cell cycle arrest in response to cadmium stress is likely to be important to allow repair and replacement of damaged DNA, proteins and lipids.

Generally, ubiquitination by SCF-type ubiquitin ligases is regulated at the level of the interaction between substrates and the substrate-adapter subunit of the ligase (e.g. Met30/Met4 interaction) [8]. Remarkably, even though Met4 ubiquitination is blocked in response to cadmium stress, the interaction between Met30 and Met4 is unchanged [12,13]. However, immunopurification experiments demonstrated that the interaction of Met30 with the SCF core component Skp1 is disrupted in cells exposed to cadmium, thus explaining inhibition of Met4 ubiquitination [12,13] (Fig. 3). Cadmium stress most likely targets Met30 and not Skp1, because the interaction of Skp1 with the F-box protein Cdc4 is unaffected by cadmium [13]. Importantly, Met4 ubiquitination *in vitro *using a reconstituted system was unaffected by addition of cadmium [13]. Furthermore, addition of yeast cell lysates prepared from cadmium-exposed cells did not affect Met4 ubiquitination in this *in vitro *reaction [13]. Together these results suggest an indirect mechanism that requires active metabolism for cadmium-induced disassembly of SCF^Met30 ^[13]. Interestingly, a similar regulation of the F-box protein/Skp1 interaction has been suggested for SCF^Grr1 ^in response to changes in growth conditions [37]. More importantly, stress-induced disassembly of SCF-type ubiquitin ligases appears to be a conserved mechanism for regulation of the transcriptional response to stress conditions. In mammals, oxidative stress induces a transcription program that depends on activation of the transcription factor Nrf2 [38-40]. Similar to Met4 in budding yeast, Nrf2 regulates glutathione levels by inducing expression of the two subunits that form mammalian γ-glutamyl-cysteine-synthase [41]. Nrf2 protein abundance is regulated by ubiquitin-dependent degradation mediated by the Cul3-based ubiquitin ligase SCF3^Keap1 ^[42-45]. In striking similarity to the budding yeast SCF^Met30^, it has been suggested that the mammalian ubiquitin ligase SCF3^Keap1 ^is disassembled during stress conditions [42]. Furthermore, immunopurification experiments indicate that the interaction between the substrate adapter Keap1 and its substrate Nrf2 is constitutive [42,46], suggesting that stress-induced dissociation of Keap1 from SCF3^Keap1 ^controls Nrf2 ubiquitination. Despite the satisfying similarity in stress-mediated regulation of ubiquitin ligase integrity in budding yeast and mammals, the SCF3^Keap1^/Nrf2 results described above warrant a note of caution, because stress-induced dissociation of Keap1 from SCF3^Keap1 ^was not detected in other studies [43,46]. Oxidation-induced conformational changes in Keap1 that lead to inhibition of the ubiquitin transfer to Nrf2 has been suggested as an alternative mechanism [46].

Cadmium clearly inactivates SCF^Met30 ^by dissociation of Met30 [12,13]. This raises the question of why cells choose to inactivate a ubiquitin ligase and thus prevent ubiquitination of all its substrates, rather than to block ubiquitination of specific substrates by regulating the ligase/substrate interaction. One can speculate that SCF^Met30 ^ubiquitinates a set of proteins that are together important for coordination of the cellular response to cadmium stress. The dissociation of Met30 from the core SCF complex would be a direct way to simultaneously block ubiquitination of several SCF^Met30 ^substrates. Alternatively, one can envision that Met30 has an additional SCF^Met30^-independent function, which is activated in response to cadmium exposure and might involve the Met4/Met30 complex. It will be important to address these questions to understand the role of signal-induced disassembly of SCF-type ubiquitin ligases.

Cadmium-induced activation of Met4 not only requires inactivation of the ubiquitination reaction but also deubiquitination of the existing pool of ubiquitinated Met4 [13]. Surprisingly, Barbey and colleagues [13] demonstrated that cadmium-induced deubiquitination of Met4 is blocked in the temperature sensitive *cdc53-1 *strain, suggesting that Met4 deubiquitination depends on the cullin Cdc53. The deubiquitinating enzyme responsible for Met4 activation has not yet been identified, but clearly plays an important role in the cellular response to cadmium and arsenic stress.

## SCF^Met30 ^a link between S-adenosylmethionine levels and cell cycle progression

Depletion of methionine, cysteine or S-adenosylmethionine (SAM) leads to Met4 activation [3,47,48]. Metabolic pathways interconnect these three compounds so that low concentrations of methionine will result in low concentrations of SAM and cysteine [3] (Fig. 1). Cysteine has recently been identified as the most terminal effector in regulation of Met4 activity [47,48] and probably connects intracellular methionine and SAM concentrations to Met4 ubiquitination [48]. We speculate that tight coordination of cell cycle progression and S-adenosylmethionine is essential and one of the important functions of SCF^Met30^. SAM is the major methyl donor in cells and provides activated methyl groups in a number of important reactions, such as phospholipid synthesis, protein methylation and synthesis of the 7-methylguanosine mRNA cap. Histone methylation is an important epigenetic mark that requires adequate SAM levels for its duplication during cell division. A similar argument can be made for DNA methylation, which is, however absent in budding yeast. It is therefore reasonable to assume that cells have developed mechanisms to prevent cell cycle progression when SAM levels are too low to support efficient methylation of histones or DNA. We therefore suggest that the SCF^Met30^/Met4-controlled cell cycle steps are components of a checkpoint pathway that ensures epigenetic stability.

## Conclusion

SCF^Met30 ^is required for several steps during the cell cycle (Fig. 1). The cell cycle functions appear to be primarily mediated by a transcription complex formed by the two proteins Met4 and Met32. When activated, the Met4/Met32 complex stops the cell cycle. SCF^Met30 ^inhibits Met4 activation by assembling a ubiquitin chain at the lysine residue in position 163 of Met4. Strong genetic evidence shows a common essential function for ubiquitin ligases from various organisms involved in stress response. The ubiquitin ligases SCF^Met30 ^(*S. cerevisiae*), SCF^Pof1 ^(*S. pombe*), and SCF3^Keap1 ^(mammals) inactivate their bZIP transcription factor targets Met4, Zip1, and Nrf2, respectively [5,30,49]. In fission yeast, Zip1 activates cell cycle arrest and Zip1 activation is suppressed by the ubiquitin ligase SCF^Pof1 ^[30]. SCF^Pof1 ^and its budding yeast relative SCF^Met30 ^are thus required for cell cycle progression. Interestingly, the postembryonic lethality of Keap1-null mice is due to hyperactivation of the transcriptional activator Nrf2 and is suppressed in compound Keap1^-/- ^Nrf2^-/- ^mutant mice [49,50]. The lethality of Keap1-null mice is however not caused by cell cycle arrest but appears to result from malnutrition [49].

Several cell cycle steps have been identified as targets of the SCF^Met30 ^cell cycle function [5,6]. But there is currently an almost complete lack of mechanistic insight into how Met4 or Zip1 inhibit cell proliferation. However, it is evident that these two bZIP transcription factors are activated in response to harmful environmental conditions such as heavy metal stress and that they activate a transcriptional response that coordinates cellular defense mechanism and cell cycle arrest. A detailed understanding of these stress-regulated ubiquitin ligases, their transcription factor targets and their cell cycle effects will be important given the impact heavy metal and oxidative stress have on carcinogenesis and aging.

## Abbreviations

bZIP: basic leucine zipper

preRC: pre replication complex

SAM: S-adenosylmethionine

SCF: Skp1_Cullin_F-box

SCF3: Cullin3-based ubiquitin ligase (also referred to as BCR)

UIM: ubiquitin interacting motif

## Competing interests

The author(s) declare that they have no competing interests.
